# Annotations

**Published:** 1905-10-14

**Authors:** 


### ANNOTATIONS.

### Professor Behring's New "Cure."

During the past week, at the closing sitting of the Tuberculosis Congress in Paris, a statement, the full importance of which only the future can reveal, was made by one of the most experienced, careful, and successful workers in scientific medicine. By research carried on in the course of the last two years, Professor Behring believes that he has discovered a new curative principle which is the chief immunising agent in his "bovovaccine." This principle, designated T.C., is present, he states, in the tubercle bacillus. In the process of immunisation against tuberculosis the T.C. is freed from accidental substances and exerts a symbiotic action in the interior of organic cells, especially those of lymphatic origin. Behring endeavours to spare the organism the work, often long and dangerous, of the elaboration of the T.C.; then follows the problem of freeing the T.C. from substances hindering its therapeutic action. These bacillary substances are classified in three groups, and after their removal what remains of the tubercle bacillus is designated "Rest bacillus." This rest bacillus, or residue bacillus, retains the form and staining qualities of the tubercle bacillus, but by suitable preparation it can be made an amorphous substance. It is the elaboration of this amorphous substance (T.C.), by the lymphatic cells, which develops the state of immunity of the organism. Behring is confident of producing T.C. in the laboratory as a remedy which can be used without danger on the human subject; he hopes thus to confer a passive immunity. It will be seen at once that there is nothing conclusive in Behring's statement, for the clinical and therapeutic value has yet to be tested; but he has undoubtedly advanced and widened the field of research in tuberculosis.

### Hygiene in Schools.

Between schooling and sanitary instruction there is, as Dr. Heron felicitously stated the matter to his audience in Paris at the same Congress, a *rapprochement*, but not yet *l'entente cordiale*, which he desires to see established, and from which he anticipates results of a highly beneficial character. He, and those who think with him, maintain that the preventible character of illness and its ordinary dependence upon dirt, upon want of ventilation, and upon improper feeding, should be impressed upon children in their early years; and they believe—or at least they hope—that a future generation may grow up less hardened in evil ways than are many of those who have at present attained maturity. It is very difficult to say to what extent school influences may reasonably be expected to correct or overpower those of a dirty home, and we are inclined to think that, in order to render the teaching effective, the educational authorities should be empowered to enforce an observance of cleanliness in the schools themselves. In some of the schools in America, we are informed, no dirty child is permitted to enter a class-room until it has had a bath, and has put on a suit of clothing provided by the authorities for the purpose, and again taken off before it returns home, the expense of this provision being charged to the parents, and being recoverable from them in a summary manner. The law is a great educator —greater even than the schoolmaster—and if children were compelled to be clean in the schools, and if parents were compelled to do all in their power to render them so, it is certain that the importance of the lessons in hygiene would be far better appreciated than it is likely to be at present.

### Christian Science and Faith-healing.

The last afternoon of the Church Congress at Weymouth, which closed on Friday, was mainly occupied by a discussion on Christian science and faith-healing. The Rev. W. S. Swayne, Vicar of St. Peter's, Cranley Gardens, who introduced it, declined to treat Christian science as the creed of fools or charlatans. He said that healing by suggestion was perfectly well understood and was deliberately practised in London with good results, and to a larger degree in Paris. At this point he elicited cheers by declaring that there are many people, especially in the middle and upper classes, who concern themselves far too much about their health. The evil, he said, was a growing one, and Christian science had done some of its adherents the truest service when it had told them that they were perfectly well if they could only believe it, and that the less they thought about their health the better. But there is no need to fly to Christian science for advice of this kind. It is, perhaps, inevitable that there should be a certain number of medical men who encourage the delusions of healthy patients that they are invalids. But the majority have neither the inclination nor the time to dance attendance on persons who have nothing the matter with them. It was an amusing commentary on Mr. Swayne's paper that one of the succeeding speakers remarked that "most people believe more in the doctor than in the parson when they are ill." We do not know how far this is accurate, but it is certain that the medical man who deals frankly with his patient, neither prescribing for imaginary ailments, nor minimising serious symptoms, is as indispensable as he is welcome in the sick-chamber. Subsequently, Mr. Swayne contended that on the physical side the failures of Christian science have been numerous and disastrous. It leaves, he insisted, no room for works of mercy, spiritual or bodily; for if its teachings are true, there is no reason why the hungry should be fed, the sick in hospitals and homes for the dying ministered to, or the outcast and the miserable cared for. Then, why coquet with it? Mr. Swayne thinks that the average convert troubles himself little "about Mrs. Eddy's absurd metaphysics or blasphemous theology," but the average convert who admits the claims of Christian science to be able to cure all diseases, not only exposes himself to the most grave risks, he also jeopardises the health of his fellow-creatures in a manner which it is impossible to justify either on grounds of science or of common sense.

